# Thermodynamic Features of Structural Motifs Formed by β-L-RNA

**DOI:** 10.1371/journal.pone.0149478

**Published:** 2016-02-23

**Authors:** Marta Szabat, Dorota Gudanis, Weronika Kotkowiak, Zofia Gdaniec, Ryszard Kierzek, Anna Pasternak

**Affiliations:** Institute of Bioorganic Chemistry, Polish Academy of Sciences, Noskowskiego 12/14, 61–704, Poznan, Poland; University of Quebec at Trois-Rivieres, CANADA

## Abstract

This is the first report to provide comprehensive thermodynamic and structural data concerning duplex, hairpin, quadruplex and i-motif structures in β-L-RNA series. Herein we confirm that, within the limits of experimental error, the thermodynamic stability of enantiomeric structural motifs is the same as that of naturally occurring D-RNA counterparts. In addition, formation of D-RNA/L-RNA heterochiral duplexes is also observed; however, their thermodynamic stability is significantly reduced in reference to homochiral D-RNA duplexes. The presence of three locked nucleic acid (LNA) residues within the D-RNA strand diminishes the negative effect of the enantiomeric, complementary L-RNA strand in the formation of heterochiral RNA duplexes. Similar behavior is also observed for heterochiral LNA-2′-O-methyl-D-RNA/L-RNA duplexes. The formation of heterochiral duplexes was confirmed by ^1^H NMR spectroscopy. The CD curves of homochiral L-RNA structural motifs are always reversed, whereas CD curves of heterochiral duplexes present individual features dependent on the composition of chiral strands.

## Introduction

Oligonucleotides have a wide range of applications related with the regulation of the biological and structural functions of nucleic acids. These molecules can be considered to be potential therapeutics involved in the modulation of pathogenic features of RNAs or DNAs. Oligonucleotides constitute an interesting alternative to currently marketed drugs that target proteins, which represent a relatively narrow group of total cellular proteins. Oligonucleotide-based drugs that target mRNA (encoding all cellular proteins) have the potential to be effective for diseases not treatable by currently used drugs. In general, oligonucleotide therapy may consist of three different approaches *i*.*e*. RNA interference (RNAi), antisense strategy and steric blocking [1]. However, recently nanotechnology has attracted more attention as a promising alternative for classic oligonucleotide-based therapies. The conjugates of oligonucleotides and nanoparticles are characterized by low immunogenicity and toxicity, increased permeability across biological membranes, and less side effects due to decreased drug dose and increased specificity of interactions with target molecules [2]. Naturally occurring DNA and RNA molecules are rapidly degraded by cellular nucleases. Thus, it is important to increase their biological stability and improve binding affinity toward complementary sequences. To this end, various chemical modifications of oligonucleotides have been developed to improve the properties of nucleic acids *e*.*g*. 2'-O-methyl-RNA [3], locked nucleic acids (LNAs) [4], 2′-fluoro-RNA [5], unlocked nucleic acids (UNAs) [6, 7], 2′-O-(2-methoxyetyl)-RNA (2′MOE) [8] or phosphorothioates [9]. Recently, several studies have focused upon nucleic acids containing unnatural L-(deoxy)ribose. L-DNA and L-RNA are molecules, where the natural D-(deoxy)ribose is replaced by the L-enantiomer and can form, upon hybridization, a left-handed double helical structure. The L-oligonucleotides are not recognized by enzymes consisting of L-amino acids. They show extraordinary resistance to biological degradation and can be considered useful therapeutic tools [10]. Recently, L-oligonucleotides have found a potential application as aptamers for inhibiting gene expression. L-RNA aptamers (Spiegelmers) were first reported more than 15 years ago for binding to D-adenosine and L-arginine [11, 12]. Furthermore, the Klussmann group demonstrated mirror-image DNA aptamers that bind and selectively inhibit glucagon [13]. A similar approach was taken by Joyce and co-workers. They developed an L-RNA aptamer that binds the natural D-form of HIV-1 trans-activation responsive (TAR) RNA [14]. Binding of an L-aptamer to a target TAR RNA inhibits formation of the Tat-TAR complex that is essential for HIV-1 replication. L-DNA was also proposed for use in antisense strategy many years ago; however, it failed to form stable hybrids with D-RNA [15, 16]. Recently, Erdmann and co-workers have investigated mirror-image hammerhead ribozymes and DNAzymes to recognize and efficiently cleave complementary enantiomeric nucleic acids [17]. These reports are mainly focused upon biological features and functions that could make the L-oligonucleotides valuable for potential therapeutic and diagnostic applications.

Thermodynamic studies published so far suggest that L-modified oligonucleotides containing all four canonical nucleotides do not hybridize to natural DNA or RNA [18, 19]. However, heterochiral duplexes are formed by homopurinic and homopyrimidinic strands [18]. Heterochiral duplex in this paper refers to two oligonucleotide strands of opposite chirality which are involved in reversible non-covalent interactions, not necessarily canonical Watson-Crick hydrogen bonding. Moreover, Ashley indicated fairly strong hybridization of [L-dU]_20_ and poly(rA) in the presence of 5 mM MgCl_2_ [20]. In general, if heterochiral duplexes are formed, they are always less stable in reference to homochiral counterparts. Urata *et al*. and Kawakami *et al*. studied the influence of single L-nucleotide residue on the stability of DNA, RNA and DNA/RNA duplexes and demonstrated that the incorporation of an unnatural residue significantly destabilizes formed structures [21–23].

In this paper, the common structural motifs formed by L-RNA were analyzed thermodynamically. Also, the quantitative effect of the inverted chirality of homopyrimidinic or homopurinic strands on the stability of heterochiral RNA duplexes was studied. The model structures were characterized by circular dichroism (CD) and nuclear magnetic resonance (NMR) spectra.

## Materials and Methods

### Oligonucleotides

The β**-**L-RNA phosphoramidite monomers were purchased from ChemGenes. All oligonucleotides were synthesized on a MerMade12 (BioAutomation) RNA/DNA synthesizer using standard phosphoramidite chemistry. Thin-layer chromatography (TLC) purification of the oligonucleotides of length 6–12 nt was carried out on Merck 60 F254 TLC plates with the 1-propanol/ammonia/water = 55:35:10 (v/v/v). The oligonucleotides of length 13–32 nt were purified by polyacrylamide gel electrophoresis. Details of the deprotection and purification of oligonucleotides have been previously described [24]. The composition of all oligonucleotides was confirmed using MALDI-TOF mass spectrometry, (Bruker Autoflex, S1 Table).

### UV-vis melting analysis

Oligonucleotides were dissolved in a buffer containing 100 mM sodium chloride, 20 mM sodium cacodylate and 0.5 mM Na_2_EDTA, pH 7.0 (hairpins); 1 M sodium chloride, 20 mM sodium cacodylate and 0.5 mM Na_2_EDTA, pH 7.0 (homochiral and heterochiral duplexes); 100 mM potassium chloride, 20 mM sodium cacodylate and 0.5 mM Na_2_EDTA, pH 7.0 (quadruplexes); 100 mM potassium chloride, 20 mM sodium cacodylate and 0.5 mM Na_2_EDTA, pH 3.4 (i-motif). Single-stranded oligonucleotide concentrations were calculated based on the absorbance measured above 80°C and extinction coefficients that were approximated with the nearest-neighbor model, using the ribotask.com website. LNA-modified and unmodified RNA and 2ʹ-O-methyl-RNA strands with identical sequences were assumed to have identical extinction coefficients [25]. The samples were denatured at 90°C for 2 min and then cooled to room temperature. The measurements were performed for nine different concentrations of each sample in the concentration range of 10^−4^–10^−6^ M, except for heterochiral duplexes. Melting temperatures of heterochiral duplexes were determined for 20 μM-sample concentrations *via* the first derivative of experimental melting curves. The measurements were performed in triplicate and the T_m_ value was obtained as an arithmetic average.

Melting curves were obtained using the UV melting method at 260 nm (duplexes, hairpins), 265 nm (i-motifs), and 295 nm (quadruplexes, i-motifs (S2 Table)) in the temperature range of 3–93°C with a heating rate of 1°C/min (duplexes) or 0.5°C/min (quadruplexes, i-motifs) [26, 27] on a JASCO V-650 spectrophotometer equipped with a thermoprogrammer. Melting curves were analyzed and the thermodynamic parameters determined by non-linear curve fitting using MeltWin 3.5 software. Melting temperatures calculated for a 10^−4^ M concentration of oligonucleotide are denoted by T_M_, and melting points for any other concentration of oligonucleotide are denoted by T_m_.

### CD spectroscopy

CD spectra were recorded on a JASCO J-815 spectropolarimeter. The oligonucleotides were dissolved in the same buffer as for UV melting studies to achieve a sample concentration of 3.0 μM. All samples were denatured at 90°C for 2 min and then slowly cooled to room temperature prior to data collection [28]. The spectra were recorded in triplicate at 5°C in the 205–320 nm wavelength range with a 1 nm data interval. The buffer spectrum was subtracted from the sample spectra.

### NMR spectroscopy

All NMR spectra were acquired on a Bruker AVANCE III 700 MHz spectrometer, equipped with a QCI CryoProbe. The β-D-GCAUGC and β-L-GCAUGC strands were dissolved in 150 mM NaCl, 10 mM Na_2_HPO_4_/NaH_2_PO_4_, 0,1 mM EDTA (pH 6.8), while the D1, D2, D3, D4, D5 and β-L-UCUUUCUCUUCU and β-D-AGAAAGAGAAGA strands were dissolved in 1 M NaCl, 5 mM Na_2_HPO_4_/NaH_2_PO_4_, 0,1 mM EDTA (pH 6.8). The solvent was H_2_O/D_2_O (9:1, v/v). The samples were annealed by heating at 90°C for 5 min and then slowly cooled down to room temperature. The 3 mm thin wall tubes were used with a final sample volume of 200 μl. The water signal was suppressed by excitation sculpting with a gradient pulse. Spectra were processed and prepared with TopSpin 3.0 Bruker Software.

## Results and Discussion

### Thermodynamics of L-RNA structural motifs

The thermodynamic studies of L-RNA duplexes were performed for isosequentional oligonucleotides. Detailed thermodynamic parameters for these duplexes in D-RNA series have previously been reported [29]. The goal of the studies was to investigate whether the nearest-neighbor model parameters obtained for natural D-RNA duplexes can also be applied to calculate the thermodynamic stability of enantiomeric L-RNA duplexes. Our studies confirmed that homochiral L-RNA duplexes are characterized by the same thermodynamic parameters, *i*.*e*. enthalpy, entropy and Gibbs free energy, as naturally occurring D-RNA counterparts (Table 1). Melting of L-RNA duplexes also demonstrated the two-state character of that process. Such an effect was expected, since, by definition, enantiomers possess the same chemical and physical properties, except for their ability to rotate plane-polarized light in opposite directions. Therefore, we conclude that the free energy parameters published by the Turner group can be applied for predictions of the thermodynamic stabilities of both D-RNA and L-RNA duplexes. Differences equal to 0.2–0.4 kcal/mol between free energy values reported by Freier *et al*. and reported herein are presumably due to the different methods used for the calculation of thermodynamic parameters.

**Table 1:** Thermodynamic parameters of model β-D-RNA and β-L-RNA duplexes and hairpins^a^.

Duplexes (5ʹ–3ʹ)	Average of curve fits				T_M_^−1^ vs. log C_T_ plots			
	−ΔH° (kcal/mol)	−ΔS° (eu)	−ΔG°_37_ (kcal/mol)	T_M_ ^b^ (°C)	−ΔH° (kcal/mol)	−ΔS° (eu)	−ΔG°_37_ (kcal/mol)	T_M_^b^ (°C)
GUCGAC	57.7±2.5	161.7±7.5	7.54±0.16	47.4	52.3±2.2	144.6±6.9	7.41±0.05 (7.09)	47.7 (45.4)
*GUCGAC*	58.1±3.7	162.8±11.3	7.59±0.17	47.6	51.1±1.2	140.7±3.9	7.43±0.02	48.0
GACGUC	58.8±2.4	164.5±7.5	7.77±0.07	48.4	57.9±1.4	161.8±4.4	7.72±0.03 (7.35)	48.3 (46.2)
*GACGUC*	59.5±3.3	166.6±10.3	7.82±0.12	48.6	56.5±0.7	157.4±2.3	7.70±0.02	48.5
GCAUGC	60.4±2.6	169.1±8.1	7.91±0.13	48.9	53.9±1.3	148.9±4.0	7.75±0.03 (7.38)	49.4 (45.7)
*GCAUGC*	60.5±1.7	169.4±5.2	7.91±0.10	48.9	57.5±1.3	160.3±4.0	7.82±0.03	49.0
GUGCAC	56.5±3.0	156.6±9.6	7.87±0.09	49.6	57.0±1.3	158.4±4.0	7.86±0.04 (7.65)	49.3 (47.6)
*GUGCAC*	57.1±2.1	158.5±6.5	7.90±0.14	49.6	51.3±1.0	140.5±3.2	7.76±0.03	50.1
CGUGCGAAUGAACGCACG^H^	58.6±1.3	167.5±3.9	6.65±0.12	76.7				
*CGUGCGAAUGAACGCACG*^H^	58.8±2.2	168.2±6.9	6.61±0.10	76.3				
GGCGCAAGCC^H^	35.3±7.2	103.0±21.2	3.38±0.75	69.8				
*GGCGCAAGCC*^H^	37.3±6.1	109.4±18.9	3.40±0.28	68.0				

a–solutions: 1 M NaCl, 20mM sodium cacodylate, 0.5 mM Na_2_EDTA, pH 7;

^H^– 100 mM NaCl, 20 mM sodium cacodylate, 0.5 mM Na_2_EDTA, pH 7;

b–calculated for 10^−4^ M oligomer concentration,

italic– β-L-RNA

values in parentheses–results from PNAS USA (1986), 83, 9373.

We also studied the thermodynamic stability of other commonly known RNA structural motifs in L-RNA series (Tables 1 and 2). Similar to L-RNA duplexes, D- and L-enantiomers of RNA hairpins also possess a comparable thermodynamic stability (Table 1). Moreover, both enantiomers of highly ordered RNA structures, *i*.*e*. quadruplexes and i-motifs, are characterized by the same melting curve profiles, showing a hypochromic effect at 295 nm and comparable thermodynamic stability within the limits of error (Table 2). Additionally, based on the dependence of T_m_ vs. concentration, L-enantiomers of natural RNA fold into quadruplex and i-motif structures with retained molecularity of folding, *i*.*e*. intramolecular i-motif and intermolecular quadruplex structure (data not shown).

**Table 2:** Thermodynamic parameters of quadruplex (^Q^) and i-motif (^i^) formation^a^.

Sequence	−ΔH° (kcal/mol)	−ΔS° (eu)	ΔG°_37_ (kcal/mol)	T_M_ (°C)
CCCUCCCUUUUCCCUCCC^i^	33.6±3.7	109.9±12.1	+0.46±0.21	32.8
*CCCUCCCUUUUCCCUCCC*^i^	35.2±2.9	115.1±9.3	+0.46±0.26	33.0
AGGAGGAGGAGGA^Q^	87.3±11.3	233.4±32.8	−14.88±1.13	73.6
*AGGAGGAGGAGGA*^Q^	96.4±2.3	260.2±6.8	−15.71±0.21	73.0

a–solution: 100 mM KCl, 20mM sodium cacodylate, 0.5mM EDTANa_2_, pH 7.0 (quadruplex) or 3.4 (i-motif),

italics– β-L-RNA.

### Thermal stability of heterochiral D-RNA/L-RNA and 2′-O-methyl-D-RNA/L-RNA duplexes

**Fig 1:**
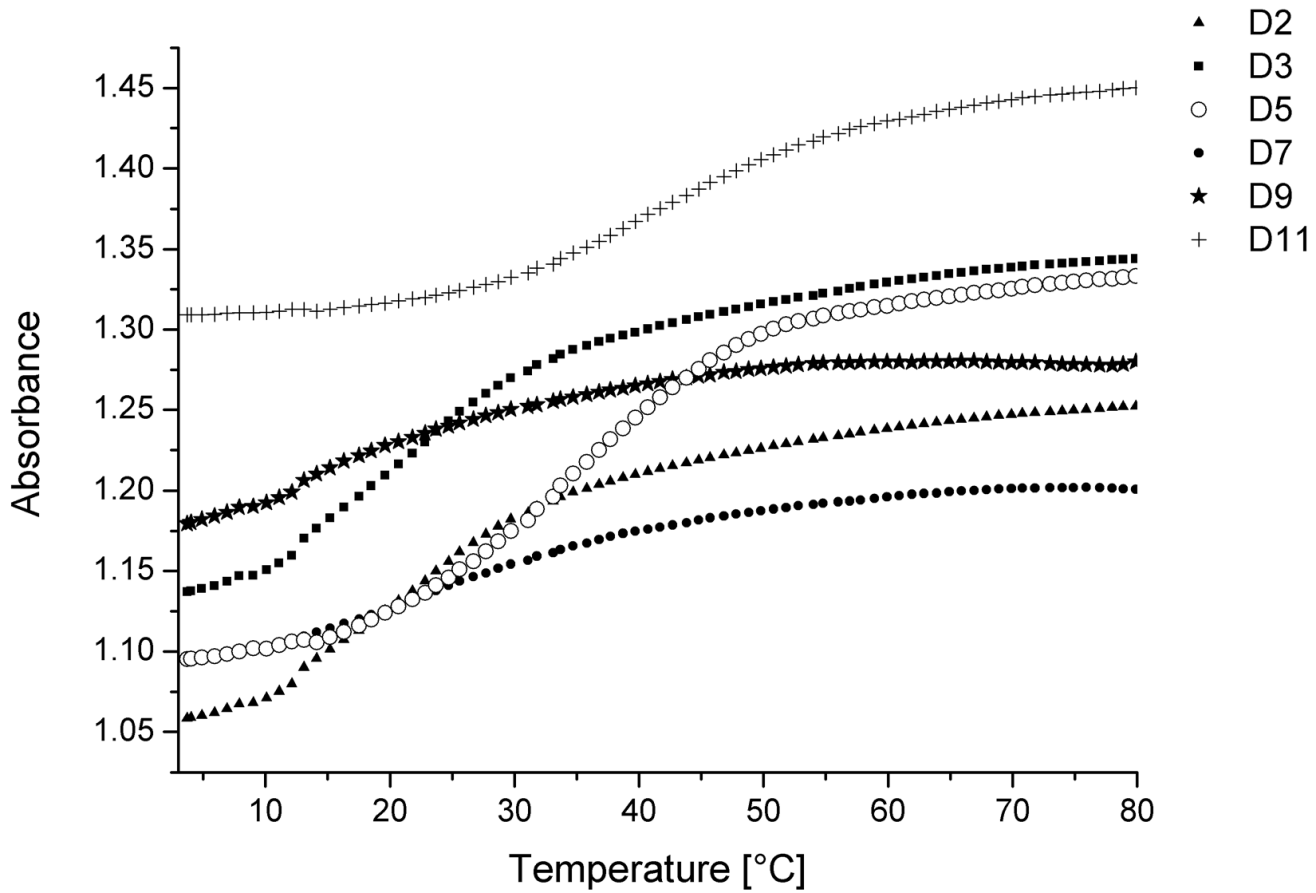
UV melting curves of model heterochiral RNA duplexes.

It has been reported that heterochiral duplexes composed of mixed-sequence homooligomers with opposite chirality cannot be formed [18, 19]. However, based on EMSA experiments and rough melting curve analyses reported so far, some hybridization may occur between homopyrimidinic and homopurinic enantiomers or 20-mer of L-dU and poly(rA) [18, 20]. We studied the quantitative effect of the inversion of chirality of 12-nt-long homopyrimidinic or homopurinic strands on the thermodynamic stability of heterochiral RNA duplexes. Unfortunately, the melting profiles of heterochiral duplexes studied herein were characterized by low cooperativity and moderately lower hyperchromicity in reference to natural D-homochiral duplexes (Fig 1). As a result, we were not able to obtain reliable thermodynamic parameters and decided to focus only on changes in melting temperatures determined for all studied variants (Table 3).

**Table 3:** Thermal stability of model heterochiral duplexes^a^.

Name	Duplexes (5ʹ–3ʹ)		T_m_^b^ (°C)	ΔT_m_^b^ (°C)
D1	AGAAAGAGAAGA	UCUUUCUCUUCU	50.0	0
D2	*AGAAAGAGAAGA*	UCUUUCUCUUCU	15.9	−34.1
D3	AGAAAGAGAAGA	*UCUUUCUCUUCU*	17.9	−32.1
D4	AGAAAGAGAAGA	U**C**^**L**^UUU**C**^**L**^UCUU**C**^**L**^U	63.6	0
D5	*AGAAAGAGAAGA*	U**C**^**L**^UUU**C**^**L**^UCUU**C**^**L**^U	41.5	−22.1
D6	AGAAAGAGAAGA	U^M^C^M^U^M^U^M^U^M^C^M^U^M^C^M^U^M^U^M^C^M^U^M^	57.6	0
D7	*AGAAAGAGAAGA*	U^M^C^M^U^M^U^M^U^M^C^M^U^M^C^M^U^M^U^M^C^M^U^M^	26.1	−31.5
D8	A^M^G^M^A^M^A^M^A^M^G^M^A^M^G^M^A^M^A^M^G^M^A^M^	UCUUUCUCUUCU	52.0	0
D9	A^M^G^M^A^M^A^M^A^M^G^M^A^M^G^M^A^M^A^M^G^M^A^M^	*UCUUUCUCUUCU*	13.5	−38.5
D10	AGAAAGAGAAGA	U^M^**C**^**L**^U^M^U^M^U^M^**C**^L^U^M^C^M^U^M^U^M^**C**^**L**^U^M^	68.7	0
D11	*AGAAAGAGAAGA*	U^M^**C**^**L**^U^M^U^M^U^M^**C**^**L**^U^M^C^M^U^M^U^M^**C**^**L**^U^M^	47.7	−21.0

a–solution: 1 M NaCl, 20 mM sodium cacodylate, 0.5 mM Na_2_EDTA, pH 7.0;

b–calculated for a 20 µM oligomer concentration;

2ʹ-OMe-RNAs are shown as A^M^, C^M^, G^M^, U^M^; LNA-C is shown as **C**^**L**^, and the beta-L-oligomers are italicized.

In general, inversion of chirality within one strand causes a serious decrease in thermodynamic stability. The thermodynamic studies were performed with oligonucleotides containing 12 nucleotides in each strand. Heterochiral RNA duplexes (Table 3, D2 and D3) are less stable by 34°C and 32°C in reference to homochiral D-RNA duplex (Table 3, D1). Inversion of the chirality of either purine or pyrimidine strand results in a similar destabilization effect. Interestingly, the presence of three LNA nucleotides within D-RNA strand diminishes the negative influence of complementary L-RNA strand. Such a modified heterochiral RNA duplex is destabilized “only” by 22°C (Table 3, D5) relative to a homochiral, LNA-modified D-RNA duplex (Table 3, D4). Additionally, we also investigated the influence of the inverted chirality of one strand within 2′-O-methyl-D-RNA/L-RNA heterochiral duplexes. Similar to D/L-RNA duplexes, the strength of the hybridization of two heterochiral strands was reduced in comparison to homochiral duplexes. Duplexes D7 and D9 were less stable by 32°C and 39°C in comparison to D6 and D8, respectively. Moreover, three LNA nucleotides introduced into the 2′-O-methyl-D-RNA strand diminished the destabilization of heterochiral duplex to 21°C (Table 3, D10 vs. D11).

### Circular dichroism spectra of homochiral RNA duplexes and heterochiral RNA duplexes

**Fig 2:**
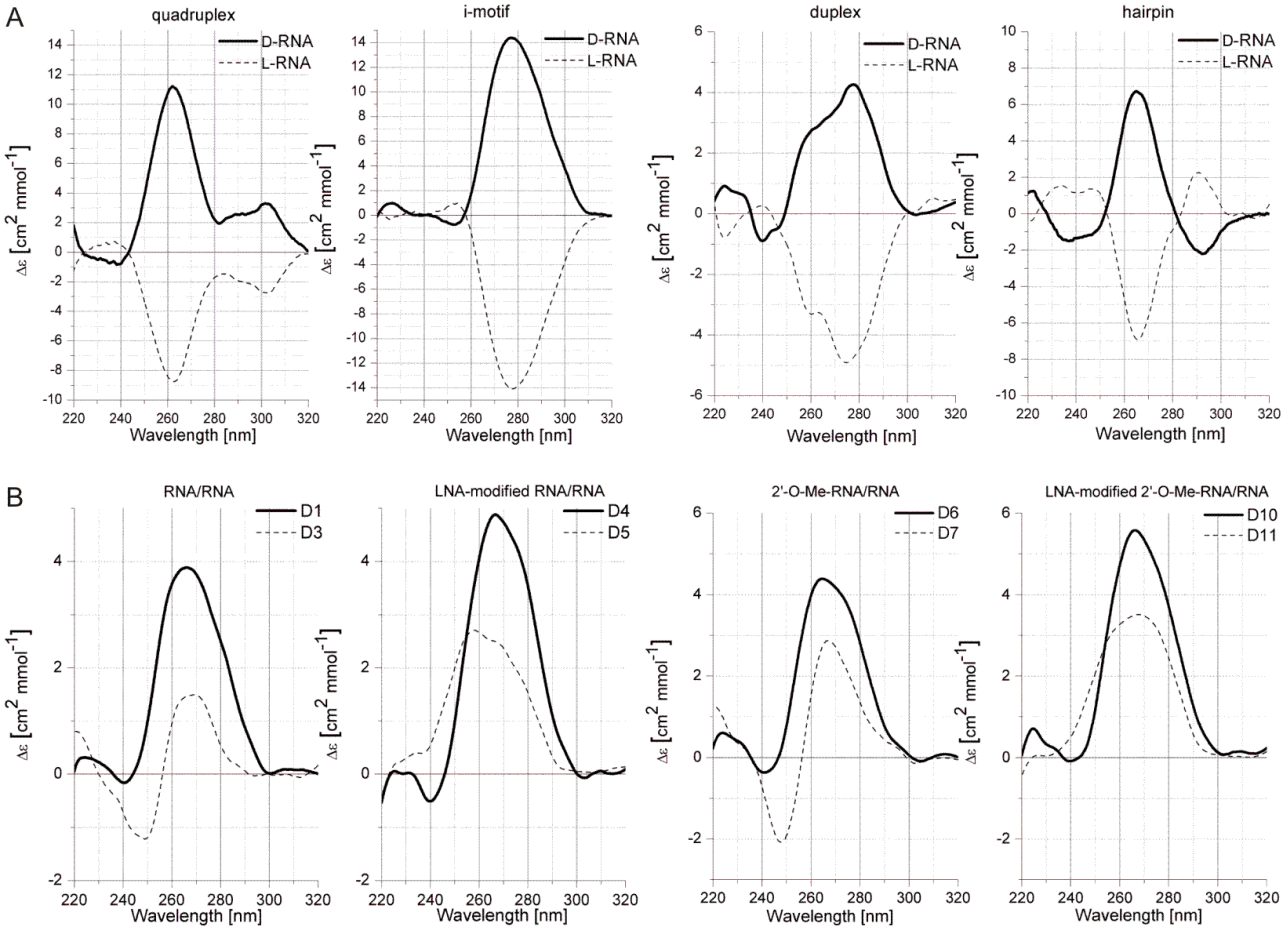
CD spectra of homochiral RNA structural motifs (A) and heterochiral duplexes (B).

Circular dichroism (CD) spectra of various enantiomeric, homochiral L-RNA structural motifs are shown in Fig 2A. In general, the patterns of CD curves are always retained but the Cotton effect is reversed and it corresponds with the definition of enantiomers. CD spectra showed that homochiral structures in L-series adopt left-handed forms. L-enantiomeric structures of duplex and hairpin are characterized by a large minimum near 280 nm and 260 nm, respectively, and a positive band of high intensity near 210 nm. CD curve of L-RNA i-motif structure possess a high-amplitude negative peak near 280 nm and a positive peak near 210 nm. The pattern of the CD curve of a quadruplex formed by L-RNA shows two high-amplitude negative peaks near 210 nm and 265 nm.

The CD spectra of heterochiral duplexes are presented in Fig 2B. The pattern of CD spectra of heterochiral RNA/RNA and LNA modified RNA/RNA duplexes, as well as heterochiral 2′-O-methyl-D-RNA/L-RNA duplex and LNA-modified 2′-O-methyl-D-RNA/L-RNA duplexes are slightly different and characteristic for each duplex. Recorded CD spectra result from individual structures formed by each heterochiral duplex. The mathematic sum of individual CD spectra of single stranded components of the heterochiral duplexes forms a different pattern (S1 Fig), thus confirming interactions between two enantiomeric strands within heterochiral duplexes. The intensity of the CD signals of heterochiral duplexes is always reduced and this is probably due to their lower thermal stability. Moreover, the maxima and minima of registered CD curves of D/L-duplexes are usually shifted toward a shorter wavelength in reference to D/D-duplexes, which might be due to the presence of additional interactions that are different from canonical Watson-Crick type. Nevertheless, positive and negative Cotton effects observed for heterochiral duplexes (at wavelengths characteristic for RNA) are similar to homochiral D-RNA duplexes regions.

### NMR spectra of homo and heterochiral RNA duplexes

We used ^1^H NMR spectroscopy to evaluate the nature of hydrogen bond pairing in the homo- and heterochiral RNA duplexes. S2 Fig shows the imino region of the representative ^1^H NMR spectra of β-L-RNA and β-D-RNA homochiral duplexes in the presence of 150 mM NaCl. The ^1^H NMR spectra of (β-D-GCAUGC)_2_ and (β-L-GCAUGC)_2_ duplexes with the chemical shifts of imino protons characteristic of Watson-Crick base pairs are identical (S2 Fig).

**Fig 3:**
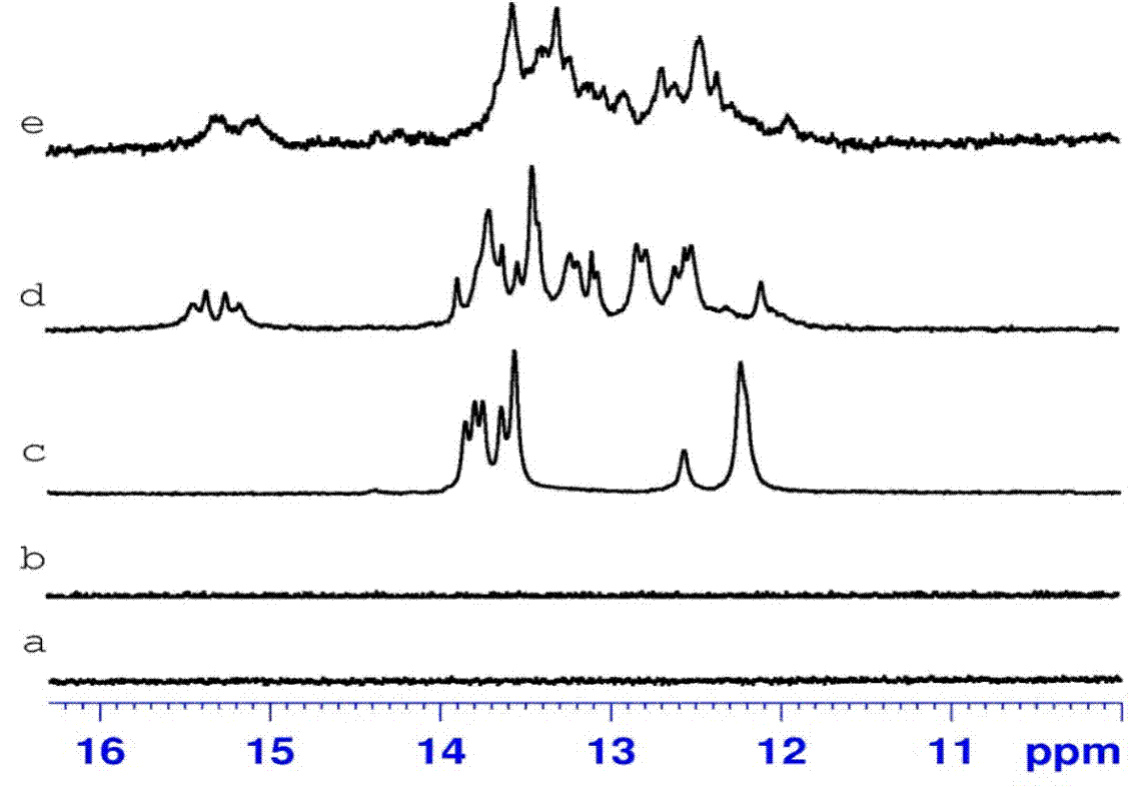
**The imino region of the ^1^H NMR spectrum of β-D-AGAAAGAGAAGA (a), β-L-UCUUUCUCUUCU (b), β-D-AGAAAGAGAAGA/β-D-UCUUUCUCUUCU (D1) (c), β-L- AGAAAGAGAAGA/β-D-UCUUUCUCUUCU (D2) (d) and β-D-AGAAAGAGAAGA/β-L-UCUUUCUCUUCU (D3) (e) recorded at 10°C in H_2_O:D_2_O (90%:10%) with 1 M sodium chloride, 5 mM phosphate buffer, pH 6.8 and 0.1 mM EDTA**.

**Fig 4:**
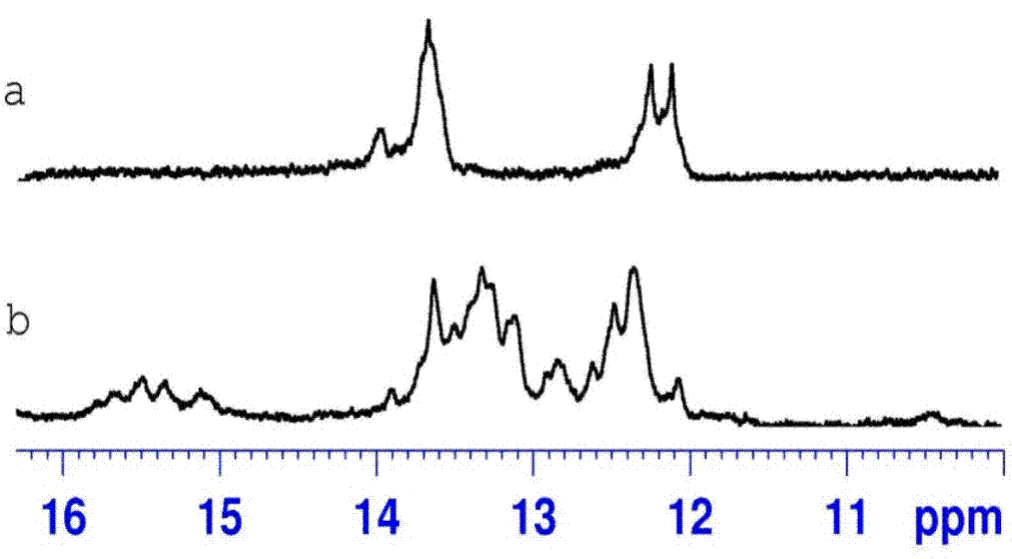
**The imino region of the ^1^H NMR spectrum of β-D-AGAAAGAGAAGA/β-D-UC^L^UUUC^L^UCUUC^L^U (D4) (a), β-^L^-AGAAAGAGAAGA/β-D-UC^L^UUUC^L^UCUUC^L^U (D5) (b) recorded at 10°C in H2O:D2O (90%:10%) with 1 M sodium chloride, 5 mM phosphate buffer, pH 6.8 and 0.1 mM EDTA**.

The absence of imino resonances in ^1^H NMR spectra of β-D-AGAAAGAGAAGA and β-L-UCUUUCUCUUCU single strands (Fig 3A and 3B) shows that no secondary structures are formed in given solution conditions (10°C, 1 M NaCl, 5 mM phosphate buffer, pH 6.8, 0.1 mM EDTA). Annealing of two homochiral strands of duplexes D1 and D4 leads to the formation of the canonical duplexes with G-C and A-U Watson-Crick base pairs (Figs 3C and 4A). A difference observed between ^1^H NMR spectra of D1 and D4 duplexes arises from the introduction of three LNA residues to D-polypyrimidine strand (β-D-UCᴸUUUCᴸUCUUCᴸU). Fig 3D and 3E present the imino region of heterochiral duplexes D2 and D3. The duplexes were obtained by annealing two heterochiral strands: β-D-UCUUUCUCUUCU and β-L-AGAAAGAGAAGA for D2 and β-L-UCUUUCUCUUCU and β-D-AGAAAGAGAAGA for D3. The presence of imino signals in the range of 11.5–16 ppm indicates the formation of the hydrogen bonds between two enantiomeric strands; however, the nature of interactions between heterochiral strands is not known. Moreover, the presence of imino resonances of different intensity indicates that in NMR conditions an equilibrium between different forms exists. Additionally, the appearance of the imino resonances above 15 ppm suggests that some cytidine residues are already protonated at pH 6.8. This implies that one of the forms in solution could be a parallel heteroduplex. The formation of heterochiral duplexes was also observed when a β-D-polypyrimidine strand was modified with three LNA cytidine residues (Fig 4B). S3 and S4 Figs compare the temperature dependence of the imino proton signals of D2 and D5, respectively. The unmodified heterochiral duplex D2 melts at a lower temperature than D5. The imino signals of D2 have already disappeared at a temperature of 35°C, whereas for an LNA-modified duplex the imino signals are visible even at 45°C. This observed increased thermal stability of the LNA modified heterochiral duplex is in accordance with the UV data, where a 20°C difference in melting temperature was determined.

## Conclusions

Here we have shown a comprehensive thermodynamic analysis of structural motifs formed by mirrored RNA. Homochiral L-RNA duplexes are characterized by the same thermodynamic parameters as their naturally occurring D-RNA counterparts. Therefore, parameters from the nearest-neighbor model that are conventionally applied for predictions of the stability of natural RNA duplexes can also be used for mirrored RNA. Thermodynamic stability of other structural motifs formed by L-RNA are also comparable, even if not always the same, to the stability of those formed by D-RNA. Heterochiral duplexes show strongly decreased thermal stability in reference to homochiral structures; however, the presence of LNA residues within D-RNA strand reduces the unfavorable energetic cost of hybridization between two enantiomeric strands. CD spectra of homochiral L-RNA structural motifs are mirror images of the curves obtained for the structures formed by D-RNA; nevertheless, the CD shape of heterochiral duplexes is similar to their homochiral D-RNA counterparts. Using NMR spectroscopy, we were able to monitor the formation of heterochiral β-D/β-L-RNA duplexes. However, in order to determine their conformational features additional study would be necessary. Unfortunately, the broadening of the majority of the resonances may hinder the use of NMR spectroscopy in further structural analyses.

## Supporting Information

S1 Fig CD spectra of homochiral (solid line) and heterochiral (dashed line) duplexes and mathematic sum of single stranded components of the heterochiral duplexes forms (dotted line). (TIF)

S2 Fig The imino c region of ^1^H NMR spectrum of β-D-GCAUGC (top) and β-L-GCAUGC (bottom) recorded at 15°C in H_2_O:D_2_O (90%:10%) with 150 mM sodium chloride, 10 mM phosphate buffer, pH 6.8 and 0.1 mM EDTA. (TIF)

S3 Fig Temperature dependence of the imino region of the ^1^H NMR spectrum of β-L-AGAAAGAGAAGA/β-D-UCUUUCUCUUCU (D2). Spectra were recorded in H_2_O:D_2_O (90%:10%) in the presence of 1 M sodium chloride, 5 mM phosphate buffer, pH 6.8 and 0.1 mM EDTA. (TIF)

S4 Fig Temperature dependence of the imino region of the ^1^H NMR spectrum of β-L-AGAAAGAGAAGA/β-D-UC^L^UUUC^L^UCUUC^L^U (D5). Spectra were recorded in H_2_O:D_2_O (90%:10%) in the presence of 1 M sodium chloride, 5 mM phosphate buffer, pH 6.8 and 0.1 mM EDTA. (TIF)

S1 Table MALDI-MS data of oligonucleotides (DOCX)

S2 Table Thermodynamic parameters of i-motif formation. a–solution: 100 mM KCl, 20 mM sodium cacodylate, 0.5 mM EDTANa_2_, pH 3.4; italic– β-L-RNA.
(DOCX)

## Abbreviations

CD: circular dichroism
NMR: nuclear magnetic resonance
