# Supplementary material for: Thermodynamic Features of Structural Motifs Formed by β-L-RNA
Source: PLoS One. 2016 Feb 23;11(2):e0149478. doi: 10.1371/journal.pone.0149478 (PMC4801053; doi:10.1371/journal.pone.0149478)
Supplement: S1 Table — (DOCX) [file pone.0149478.s005.docx]

**S1 Table MALDI-MS data of oligonucleotides**

| ***β-L-RNA sequence (5′-3′)*** | ***Calculated oligonucleotide mass*** | ***MALDI-MS [M-H]^-^ m/z*** |
| --- | --- | --- |
| GUCGAC | 1874.2 | 1873.8 |
| GACGUC | 1874.2 | 1875.7 |
| GCAUGC | 1874.2 | 1874.1 |
| GUGCAC | 1874.2 | 1874.4 |
| UAGAGAGAGAAAGUUUCGACUUUCUCUCUCUA | 10171.1 | 10164.0 |
| AGGAGGAGGAGGA | 4345.7 | 4349.1 |
| CGUGCGAAUGAACGCACG | 5793.6 | 5795.4 |
| CCCUCCCUUUUCCCUCCC | 5437.2 | 5433.4 |
| GGCGCAAGCC | 3198.0 | 3200.2 |
| AGAAAGAGAAGA | 3952.5 | 3956.4 |
| UCUUUCUCUUCU | 3608.1 | 3610.3 |
| ***β-D-RNA sequence (5′-3′)*** | ***Calculated oligonucleotide mass*** | ***MALDI-MS [M-H]^-^ m/z*** |
| GUCGAC | 1874.2 | 1875.2 |
| GACGUC | 1874.2 | 1874.8 |
| GCAUGC | 1874.2 | 1875.3 |
| GUGCAC | 1874.2 | 1874.6 |
| UAGAGAGAGAAAGUUUCGACUUUCUCUCUCUA | 10171.1 | 10168.9 |
| AGGAGGAGGAGGA | 4345.7 | 4348.8 |
| CCCUCCCUUUUCCCUCCC | 5437.2 | 5437.4 |
| CGUGCGAAUGAACGCACG | 5793.6 | 5793.2 |
| GGCGCAAGCC | 3198.0 | 3200.1 |
| AGAAAGAGAAGA | 3952.5 | 3954.5 |
| UCUUUCUCUUCU | 3608.1 | 3611.4 |
| A^M^G^M^A^M^A^M^A^M^G^M^A^M^G^M^A^M^A^M^G^M^A^M^ | 4120.9 | 4122.1 |
| U^M^C^M^U^M^U^M^U^M^C^M^U^M^C^M^U^M^U^M^C^M^U^M^ | 3776.4 | 3778.2 |
| U**C^L^**UUU**C^L^**UCUU**C^L^**U | 3686.2 | 3689.6 |
| U^M^**C^L^**U^M^U^M^U^M^**C^L^**U^M^C^M^U^M^U^M^**C^L^**U^M^ | 3812.5 | 3812.5 |
