# Supplementary material for: Thermodynamic Features of Structural Motifs Formed by β-L-RNA
Source: PLoS One. 2016 Feb 23;11(2):e0149478. doi: 10.1371/journal.pone.0149478 (PMC4801053; doi:10.1371/journal.pone.0149478)
Supplement: S2 Table — a–solution: 100 mM KCl, 20 mM sodium cacodylate, 0.5 mM EDTANa2, pH 3.4; italic– β-L-RNA. (DOCX) [file pone.0149478.s006.docx]

**S2 Table Thermodynamic parameters of i-motif formation^a^.** a – solution: 100 mM KCl, 20 mM sodium cacodylate, 0.5 mM EDTANa_2_, pH 3.4; italic – β-L-RNA

**S2 Table** Thermodynamic parameters of i-motif formation^a^.

| Sequence (5'-3') | Average of curve fits - **265 nm** | | | |  | Average of curve fits - **295 nm** | | | |
| --- | --- | --- | --- | --- | --- | --- | --- | --- | --- |
|  | **-ΔH˚ (kcal/mol)** | **-ΔS˚ (eu)** | **ΔG˚_37_ (kcal/mol)** | **T_M_ (˚C)** |  | **-ΔH˚ (kcal/mol)** | **-ΔS˚ (eu)** | **ΔG˚_37_ (kcal/mol)** | **T_M_ (˚C)** |
| CCCUCCCUUUUCCCUCCC | 33.6±3.7 | 109.9±12.1 | 0.46±0.21 | 32.8 |  | 33.7±7.6 | 110.2±25.0 | 0.54±0.22 | 32.1 |
| *CCCUCCCUUUUCCCUCCC* | 35.2±2.9 | 115.1±9.3 | 0.46±0.26 | 33.0 |  | 32.7±2.9 | 106.8±9.6 | 0.42±0.07 | 33.1 |

a – solution: 100 mM KCl, 20 mM sodium cacodylate, 0.5 mM EDTANa_2_, pH 3.4; italic – β-L-RNA
